# Case Report and Literature Review: Pulmonary Sclerosing Pneumocytoma With Multiple Metastases Harboring *AKT1* E17K Somatic Mutation and *TP53* C176Y Germline Mutation

**DOI:** 10.3389/fmed.2021.655574

**Published:** 2021-09-08

**Authors:** Qiushi Wang, Chunlin Lu, Minrui Jiang, Mengxia Li, Xiao Yang, Lei Zhang, Yong He, Chengyi Mao, Ping Fu, Ying Yang, Hualiang Xiao

**Affiliations:** ^1^Department of Pathology, Daping Hospital, Army Medical University, Chongqing, China; ^2^Department of Radiology, Daping Hospital, Army Medical University, Chongqing, China; ^3^Cancer Center, Daping Hospital, Army Medical University, Chongqing, China; ^4^Genecast Precision Medicine Technology Institute, Beijing, China; ^5^Department of Respiratory Disease, Daping Hospital, Army Medical University, Chongqing, China

**Keywords:** pulmonary sclerosing pneumocytoma, metastasis, *AKT1*, *TP53*, whole-exome sequencing

## Abstract

Pulmonary Sclerosing Pneumocytoma (PSP) is considered as a benign tumor, although a few cases have been reported to have multiple lesions, recurrence, and even regional lymph nodes (LNs) metastasis. Here, we report a case of PSP with atypical histologic features and malignant biological behavior, and explore its molecular genetic changes. The 23-year-old male showed a 6.5-cm pulmonary nodule in the right middle lobe (RML) and enlarged media stinal LNs. He underwent thoracoscopic RML lobectomy, systematic LNs dissection, and mediastinal lymphadenectomy. The metastases to the cervical LNs and liver were detected in a short period and then resected. Postoperative pathological examination confirmed the diagnosis of PSP in all the lesions, based on the histological characteristics and immune phenotypes. Furthermore, whole-exome sequencing identified both *AKT1* E17K somatic mutation and *TP53* C176Y germline mutation in this case. Thus, we presented an extremely rare case of atypical PSP with rapid recurrence and multiply metastases, which can easily be misdiagnosed as primary lung cancer. In addition, PSP-specific *AKT1* E17K somatic E17K somatic mutation accompanied with *TP53* C176Y germline mutation may contribute to the malignant clinical course of this tumor.

## Introduction

Pulmonary sclerosing pneumocytoma (PSP), formerly known as pulmonary sclerosing hemangioma, is a rare primary lung tumor originated from incompletely differentiated type II pneumocytes. Although PSP has some clinical and imaging characteristics for differentiation from similar lesions, these are not specific for diagnosis (1, 2). At present, its diagnosis still depends on surgical pathology. Occasionally, PSP may manifest as multiple lesions, recurrence, regional lymph nodes (LNs), or single organ metastasis, but is not likely to affect the prognosis. Therefore, PSP is considered as a benign or a low-grade malignant potential primary lung tumor (3–8). Herein, we report an extremely rare PSP case with atypical histological features, rapid recurrence and multiply metastases, and try to elucidate the molecular mechanism of the malignant progression through clinic pathological, Immunohistochemistry (IHC), and whole-exome sequencing (WES) data.

## Case Presentation

### Clinical History and Examination

**Figure 1:**
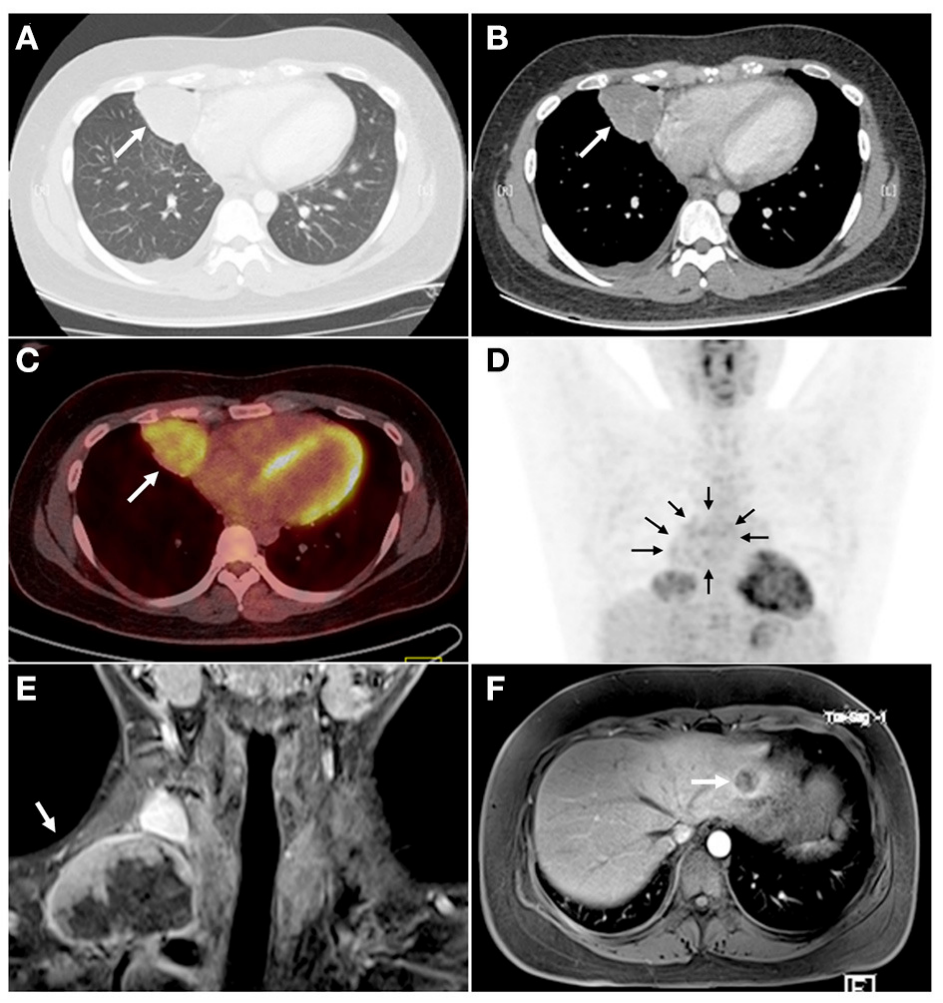
CT, PET, and MRI scans showed a primary lesion in the right lung and metastatic tumors in mediastinum, right neck, and right liver. **(A)** The lung window of CT examination showed a round-like mass (arrow) in the medial segment of the right middle lobe with a smooth margin. **(B)** The lesions (arrow) were slightly enhanced, and the tortuous small vessel shadow was seen inside. **(C,D)** PET examination showed that the radioactivity uptake of the right pulmonary pericardial lesions was increased (**C**, arrow), and the suspected annular radioactivity uptake in the mediastinum (**D**, arrows) was increased. No obvious abnormal radioactivity uptake was seen in other organs. **(E)** The neck MRI showed metastatic tumors (arrow) in the right neck after 5 months of the operation (thoracoscopic assisted resection of the right middle lung and systemic lymphadenectomy). The lesion was uneven enhancement and had abundant necrotic areas. **(F)** The abdomen MRI showed a lesion in the left lateral lobe of the liver (arrow). CT, chest enhanced computed tomography; PET, positron-emission tomography; MRI, magnetic resonance imaging.

A 23-year-old male was referred to Daping Hospital, Army Medical University for non-productive cough, chest distress, and fever for 1 month. He had no smoking and family disease history. Chest enhanced computed tomography (CT) scans confirmed a 6.5-cm pulmonary nodule in the right middle lobe (RML), with enlarged media stinal LN (Figures 1A,B). Positron-emission tomography (PET) showed increased uptake in the nodule and confirmed multiple media stinal LNs (Figures 1C,D), with no other areas of uptake. The blood examination showed that carcino embryonic antigen (CEA 8.4 ng/mL, the normal range 0.0–5.0 ng/mL) and CA153 (274.55 U/mL, the normal range 0.0–31.3 U/mL) were abnormal. The transthoracic radiology-guided biopsy considered epithelial tumor, preferred as adenocarcinoma. The patient underwent thoracoscopic RML lobectomy and systematic LNs dissection on July 27, 2018. Four months after the initial operation, the patient presented productive bloody cough. Chest enhanced CT scans showed an upper paratracheal LN measuring 3.2 × 2.2 cm. The second thoracoscopic media stinal lymphadenectomy was performed on November 20, 2018. On February 2019, 3 months after the second operation, neck-enhanced magnetic resonance imaging (MRI) showed an enlarged right supraclavicular LN measuring 5.4 × 3.8 cm (Figure 1E). The abdominal enhanced MRI showed a 1.5 × 1.2 cm nodule in the upper left lobe of the liver (Figure 1F). The patient underwent right neck mass resection on February 27, 2019, and laparoscopic liver mass resection on March 27, 2019.

### Histopathologic Findings

**Figure 2:**
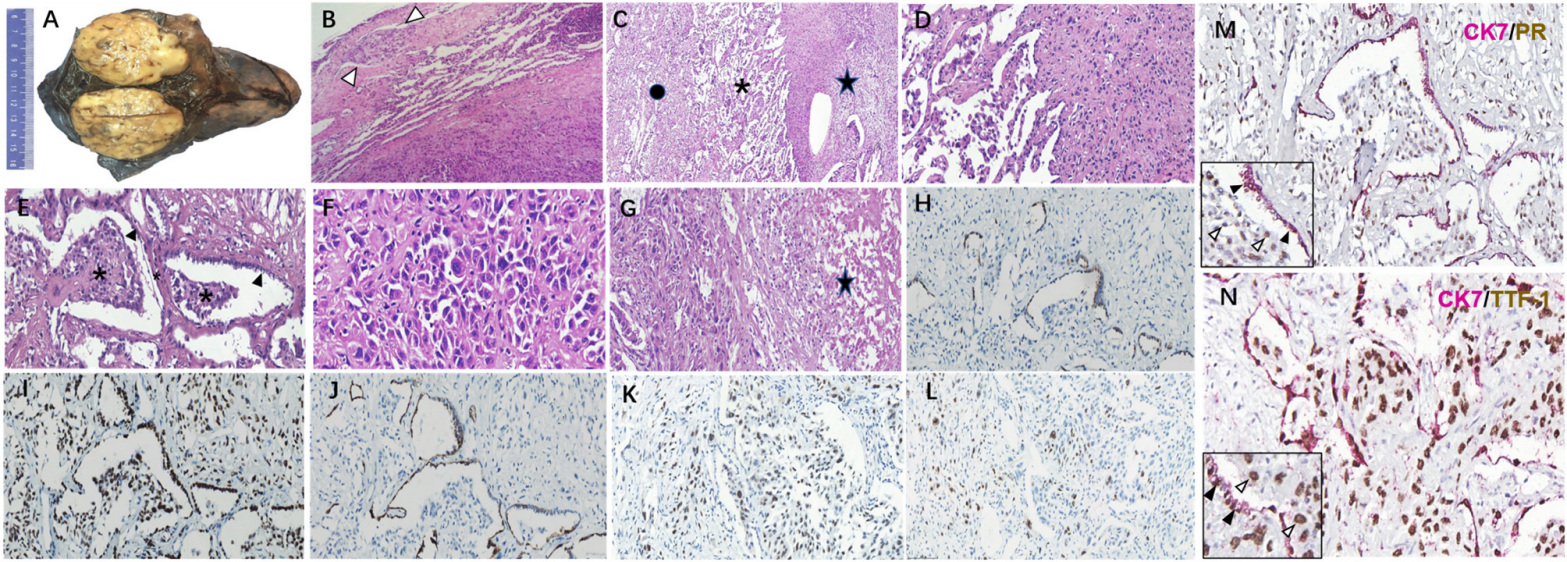
Histopathology of the primary pulmonary lesion in this unusual PSP case. **(A)** A large solid mass in the middle right lobe with relatively clear boundary and grayish-white, slightly tough texture cut surface. **(B)** The boundary between tumor and normal lung tissue was relatively clear, with the invasion of minor tumor nodules (arrow). **(C)** Solid cell area (star), papillary structure (asterisk) and sclerotic area (spot) in turn beneath normal lung tissue. **(D)** The migration of solid cell zone and papillary structures. **(E)** Papillary structures of different size (asterisk) covered with surface epithelial cells (arrow) appear in sclerotic background. **(F)** In the solid cell area, polygonal tumor cells were mainly diffusely arranged, certain atypia and giant tumor cells could be seen, but mitosis was hardly to be found. **(G)** Focal tumoral necrosis could be seen (star). CK **(H)**, TTF-1 **(I)**, and NapsinA **(J)** expressed in the same region as illustrated in this figure **(E)**. P53 **(K)** and Ki-67 **(L)** expressed in the solid cell region and papillary structures as illustrated in this figure **(F)**. **(M)** Double-labeling IHC showed the surface epithelial cells (black arrow) positive for CK7 and negative for PR, while the stromal-like cells (white arrow) in papilla's on the contrary. **(N)** Double labeling IHC showed CK7 and TTF-1 co-expressing in surface-lining cells (black arrow), while only TTF-1 positive in stromal-like cells (white arrow).Original magnification × 40 **(B–E, G–M)**, original magnification×100 **(F,N)**. PSP, pulmonary sclerosing pneumocytoma; IHC, immunohistochemistry.

A well-demarcated and circular mass, with grayish-white, slightly tough texture cut surface, was found in the RML (Figure 2A), 6 cm in diameter. Microscopically, the boundary between the tumor and the surrounding lung tissue was relatively clear except that a few minor infiltrative nodules were observed outside the main body of the tumor (Figure 2B). Solid cell area, papillary structure and sclerotic area composed of different proportion of surface epithelioid cells and polygonal stromal cells constituted the common mixed growth pattern of PSP (Figure 2C). Transition zone of solid and papillary pattern (Figure 2D), as well as the papillary structures in sclerosing background (Figure 2E) were observed, respectively. Focally in solid cell area, polygonal tumor cells distributed diffusely, with the increased cell density, and some pleomorphism and atypia appeared, but the mitosis was difficult to be found (Figure 2F). Focal tumor necrosis was noticed (Figure 2G). IHC analysis revealed that both the stromal cells and cuboidal surface cells showed positive for EMA and TTF-1 (Figure 2I), whereas only the surface cells expressed CK (Figure 2H), CEA, and Napsin A (Figure 2J). Most of the tumor cells were p53-positive (Figure 2K). The Ki-67 labeling index (LI) was about 5% (Figure 2L). PR was positive in polygonal stromal cells, while ER and Syn were positive in a few tumor cells. S-100, actin, desmin, HMB45, p63, and TFE3 were all negative. Double-labeling IHC showed CK7 and TTF-1 positive but PR negative in surface-lining epithelial cells, while CK7 negative but TTF-1 and PR positive in polygonal stromal cells (Figures 2M,N).

**Figure 3:**
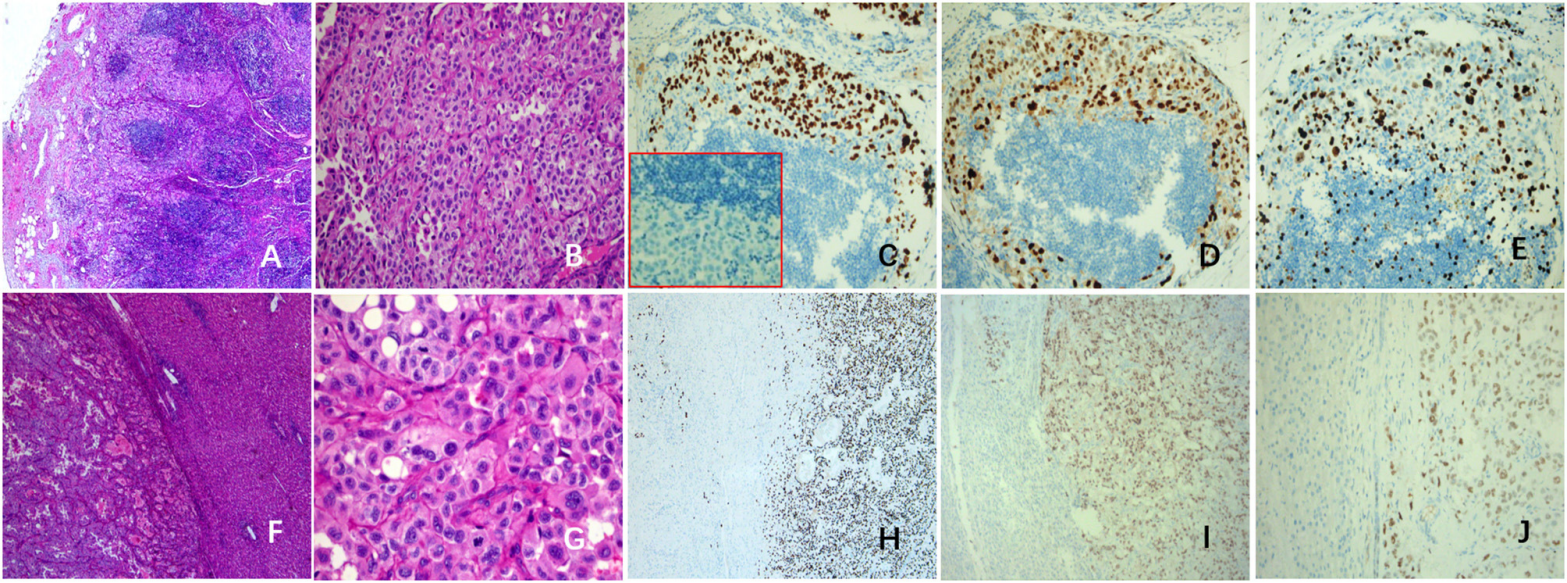
Histopathological changes in metastatic lesions of this case. Tumors cells of mediastinal LNs **(A,B)** were positive for TTF-1 **(C)** and PR **(D)**, but negative for NapsinA (**C**, red box). The Ki-67 LI was about 25% **(E)**. The tumor cells of liver metastasis **(F,G)** were like the polygonal stromal cells in the primary pulmonary lesion, with obvious pleomorphism and atypia, and the mitosis is easily to be found **(G)**. The tumor cells were positive for TTF-1 **(H)**, PR **(I)**, and P53 **(J)**. LNs, lymph nodes; LI, labeling index.

The histopathological and IHC staining of the resected specimens confirmed multiple organ metastasis of hilar, mediastinum (Figures 3A–E), cervical LNs, and liver (Figures 3F–J). Strikingly, the normal structures of LNs and the liver were partially destroyed. The polygonal tumor cells showed diffuse or patchy infiltration, increased pleomorphism and atypia, with intra-nuclear inclusions and tumor giant cells. Interstitial vascular hyperplasia and focal necrosis were easily identified. The metastatic tumors showed same immuno-phenotypes as the stromal cells of the primary lung tumor: diffuse expression of EMA, TTF-1(Figures 3C,H), PR (Figures 3D,I) and p53 (Figure 3J), but negative for CK7 and Napsin A (Figure 3C, red box). The Ki-67 LI of mediastinum LNs (Figure 3E) and liver metastasis were higher than that of the primary lesion (25 vs. 5%).

### Identification of Oncogenic Mutations of *AKT1* and *TP53*

Herein, we conducted a comprehensive examination of genetic alterations (somatic mutations) in formalin fixed paraffin-embedded (FFPE) samples from the primary and three metastatic lesions with matched adjacent normal tissue. High frequencies of *AKT1* E17K mutations was detected in primary lung tumor, media stinal LN, cervical LN, and liver metastatic lesions (16.14, 16.83, 34.51, and 21.88%, respectively). *TP53* C176Y mutations were identified in the primary lung lesion, mediastinal LN, cervical LN, and liver metastatic samples, with the frequencies of 42.41, 68.63, 72.84, and 50.52%, respectively. Interestingly, *TP53* C176Y mutations were also detected in the paired normal tissue adjacent to the primary lung tumor (frequency 33.02%), suggesting that *TP53* C176Y was a germline mutation in this patient (Table 1). This phenotype was further confirmed by the identification of the same mutation in the peripheral blood mononuclear cell (PBMC) sample.

**Table 1:** Clinical data, *AKT1*, and *TP53* gene mutations in this atypical pulmonary sclerosing pneumocytomacase.

**Location**	**Sample No**.	**Chr**	**Type**	**Freq (%)**	**Gene**	**Mutation_c**	**Mutation_p**	**Type_specific**
Lung	1820610_3	chr14	SNV	16.14	AKT1	c.49G>A	p.E17K	Missense
Mediastinal LNs	1832384_3	chr14	SNV	16.83	AKT1	c.49G>A	p.E17K	Missense
Cervical LNs	1905401_2	chr14	SNV	34.51	AKT1	c.49G>A	p.E17K	Missense
Liver	1909075_2	chr14	SNV	21.88	AKT1	c.49G>A	p.E17K	Missense
Normal tissue	1820610_3a	chr17	SNV	33.02	TP53	c.527G>A	p.C176Y	Missense
Lung	1820610_3	chr17	SNV	42.41	TP53	c.527G>A	p.C176Y	Missense
Mediastinal LNs	1832384_3	chr17	SNV	68.63	TP53	c.527G>A	p.C176Y	Missense
Cervical LNs	1905401_2	chr17	SNV	72.84	TP53	c.527G>A	p.C176Y	Missense
Liver	1909075_2	chr17	SNV	50.52	TP53	c.527G>A	p.C176Y	Missense

*LNs, lymph nodes; Chr, chromosome; Freq, frequency; SNV, single nucleotide variation*.

### Clinical Outcome After the Fourth Operation

The patient received anti-estrogen therapy with medroxyprogesterone after the fourth operation, but the disease progressed rapidly. In May 2019, chest and abdominal enhanced CT scans showed multiple nodules in the right lung and multiple enlarged LNs in neck, jaw and right supraclavicular fossa. In September 2019, the patient felt that the neck mass was significantly larger than before. Chest and abdominal enhanced CT scans showed an enlarged mass in both lung hilum (5.8 × 5.4 cm) and right supraclavicular fossa (5.7 cm in maximum dimension), multiple nodules in the liver and left kidney, and thoracic 8th and 11th vertebral osteolytic bone destruction. The patient was then administered pembrolizumab treatment at 2 mg/kg intravenously every 3 weeks, combined with apatinib at 250 mg/day orally since October 2, 2019. As of January 10, 2021, after multiple cycles of combined treatment, the metastatic lesions were obviously reduced or in a stable state. The patient was in good mental state and had no significant change in weight.

## Discussion

Due to the lack of specificity in clinical and imaging features, the diagnosis of PSP mainly depends on postoperative pathological examination. The diagnosis of typical PSP, characterized by two different cell components, four typical histological types, and immune phenotypes with specific features, is not difficult for pathologists (9). However, some atypical cases may exhibit either different clinical behaviors from typical PSP, such as recurrence or metastasis, or confused histological characteristics, such as limited typical patterns, cytological atypia, and focal necrosis. These atypical cases could be misdiagnosed as papillary or solid subtype of lung adenocarcinoma and neuroendocrine tumor, especially in the case of needle biopsy or intraoperative frozen diagnosis (10). In this case, the delayed diagnosis at biopsy was mainly attributed to the atypical phenomenon, including multiple LN metastases, papillary and solid growth pattern, cellular atypia, tumoral necrotic foci, and higher Ki-67 LI than that of ordinary PSP cases. Interestingly, only interstitial round cells, no surface cells, in LN and liver metastases of this case were observed. Consistently with other reports of the literature (3, 5, 11, 12), this morphology indicated that the stromal cells might play a critical role in malignant progression of PSP. Therefore, for accurate conclusions, we should comprehensively analyze the results of imaging, gross specimen performance, histopathology, IHC, and molecular detection, if necessary.

There are several points worth emphasizing on the pathological diagnosis. Firstly, PSP usually presents as an isolated, solid, and well-defined mass, which is different from the general changes of invasive adenocarcinoma. Secondly, we should deduce the basic structure for the diagnosis of PSP by observing the sections carefully and comprehensively: the papillary growth pattern and its diffuse distribution of interstitial cell components, as well as other histological patterns, such as solid cell area, intra-alveolar hemorrhage, and sclerotic changes. Thirdly, although there is no specific single antibody for diagnosis, establishing the diagnosis by the appropriate combination of antibodies and observing the obvious difference of immunophenotypes between epithelioid cells and stromal cells is imperative. Finally, for the atypical cases, the molecular pathologic findings are helpful for diagnosis. As in this case, we identified the *AKT1* E17K point mutation through WES analysis, which is a relatively specific molecular feature of PSP (13, 14), but no other common driving gene mutations related to lung cancer were found, which played an important role in strengthening our confidence in the diagnosis of this atypical disease.

PSPs were mostly diagnosed in female patients (83.34%), aged 38–61 years (15). The malignant progression and metastasis of PSP were extremely rare. The reported PSP patients with LN (3, 7, 8, 16) or organ metastases (5, 11) were mostly females, and only one male patient suffered from mediastinal LN metastasis (17). Herein, we reported an extremely atypical case: a young male patient had been suffering from an aggressive PSP with multiple LN and organ metastases during 7 months after the resection of the primary lung lesion.

*AKT1* E17K mutation, identified from all primary and metastatic lesions in this PSP case, were localized to the pleckstrin homology domain (PH domain), which is crucial for membrane localization and downstream activation of *AKT1* (18) and is known to promote growth factor-independent cell proliferation (19, 20). However, though more than 40 PSP cases have been reported with *AKT1* E17K mutation (13, 14), malignant progression has been rarely reported, which indicated that single E17K mutation on the PH domain of the *AKT1* gene might not be sufficient to initiate the malignant transformation of the tumor. A 17-year-old girl suffering from multiple nodules in the right lung lobe diagnosed as PSP with both *AKT1* E17K and *BRAF* V600E mutations (21). The other PSP case with diffusely scattered nodules in the right lung, harbored *AKT1* E17K and other 14 somatic gene mutations (22). These indicated that the combination of *AKT1* mutations with other oncogenes might accelerate the malignant progression of benign PSPs. For the first time, we reported a *TP53* C176Y germline mutation, a likely pathogenic mutation according to the ClinVar and 1000Genomes database, in this extremely aggressive PSP case. Which consistent with the positive expression of the P53 mutant protein in the IHC assay. Therefore, rapid malignant progression leading to multiple metastases of this PSP case might be partially attributed to the combination of somatic *AKT1* E17K mutation and germ line *TP53* C176Y mutation. Germ line mutations of *TP53* gene have been identified in 80% of patients with Li-Fraumeni syndrome (LFS), a cancer predisposition syndrome associated with high risks for a diverse spectrum of childhood- and adult-onset malignancies (23). However, neither first- nor second-degree relatives had been diagnosed with any cancer or sarcoma, this patient does not meet the classic LFS diagnosis criteria (24).

## Conclusion

We report an extremely rare case of PSP, which showed obvious atypical features in histopathology and malignant biological behaviors, such as rapid recurrence and multiple metastases. Based on the results of WES, we speculated that the somatic *AKT1* E17K and germline *TP53* C176Y mutations might account for this malignant progression.

## Data Availability Statement

All data sets generated for this study are included in the article/supplementary material, further inquiries can be directed to the corresponding author.

## Ethics Statement

The studies involving human participants were reviewed and approved by the Clinical Ethics Committee of Daping Hospital, Army Medical University (Chongqing, China). The patients/participants provided their written informed consent to participate in this study. Written informed consent was obtained from the individual(s) for the publication of any potentially identifiable images or data included in this article.

## Author Contributions

QW and HX designed the study. MJ and XY investigated and provided the clinical data. QW, CL, LZ, and YY performed the molecular experiments. CM, CL, and PF performed the pathologic slides. QW and HX wrote the manuscript. ML, YH, and HX revised and edited the manuscript. All authors contributed to the article and approved the submitted version.

## Funding

This work was supported by The National Natural Science Foundation of China (grant no. 81802781).

## Conflict of Interest

The authors declare that the research was conducted in the absence of any commercial or financial relationships that could be construed as a potential conflict of interest.

## Publisher's Note

All claims expressed in this article are solely those of the authors and do not necessarily represent those of their affiliated organizations, or those of the publisher, the editors and the reviewers. Any product that may be evaluated in this article, or claim that may be made by its manufacturer, is not guaranteed or endorsed by the publisher.
